# Coronary Guidewires in Temporary Cardiac Pacing and Assessment of Myocardial Viability: Current Perspectives and Future Directions

**DOI:** 10.3390/jcm12226976

**Published:** 2023-11-08

**Authors:** Rabeia Javid, Nancy Wassef, Stephen B. Wheatcroft, Muzahir H. Tayebjee

**Affiliations:** 1 Leeds Institute of Cardiovascular and Metabolic Medicine, School of Medicine, University of Leeds, Leeds LS2 9JT, UK; rabeia.javid@nhs.net (R.J.); s.b.wheatcroft@leeds.ac.uk (S.B.W.); 2 Leeds General Infirmary, Department of Cardiology, Leeds Teaching Hospitals NHS Trust, Great George Street, Leeds LS1 3EX, UK; nancy.wassef1@nhs.net

**Keywords:** myocardial viability, trans coronary, intracoronary, pacing

## Abstract

Intracoronary guidewires used in percutaneous coronary intervention can also be configured to provide temporary ventricular pacing. Trans coronary electrophysiological parameters recorded by employing coronary guidewires may have a potential role in assessing myocardial viability and could provide a means to make an immediate on-table decision about revascularisation. To date, some small studies have demonstrated the safety of this technique in temporary cardiac pacing, but further research is required to refine this approach and establish its clinical utility in myocardial viability assessment. In this review we discuss the potential role of trans coronary electrophysiology in the assessment of myocardial viability.

## 1. Introduction

Coronary artery guidewires are essential in percutaneous coronary interventions (PCIs) by enabling the over-the-wire delivery of balloons, stents, imaging probes and mechanical devices to the target vessel. Pacing via a coronary guide wire was first attempted in the 1980s [1] and more recently trans coronary pacing has been studied as a potential method to assess myocardial viability [2]. In this article, we review the evidence that supports the use of coronary guide wires for pacing and discuss whether electrophysiological parameters recorded via coronary guide wires can be utilised to assess myocardial viability in the catheter laboratory. In this review the term trans coronary will be applied to the electrophysiological parameters recorded as these involve the placement of additional electrodes outside the coronary artery (skin electrodes), as opposed to intra coronary which refers to the anatomical placement of a wire in the coronary artery.

## 2. Literature Search

The following databases were used for the literature review: Medline, Embase, PubMed and CINHAL. A combination of medical subject headings (MeSH) included the following: coronary pacing, trans coronary pacing, intracoronary pacing, Ic pacing, guide wire pacing, LV pacing, cardiac pacing, left heart pacing, emergency pacing, viability, myocardial viability, cardiac viability, cardiac imaging, nuclear imaging and cardiac MRI. The literature search was limited to studies published in the English language up until 2023. Titles and abstracts were screened for suitability to be included. The references cited in each eligible study were considered for additional citations. The final literature search was performed in June 2023.

## 3. Coronary Guidewires

Coronary guidewires are composed of a central core, a radio-opaque distal tip and a covering or coating which facilitates delivery and lesion crossing. The central core is typically stainless steel or nitinol (nickel–titanium alloy) and is in physical and electrical continuity with the flexible distal tip. Therefore, coronary guidewires can conduct electricity from the proximal end of the wire all the way to the distal tip, which is intravascular and often in contact with the coronary endotheium. The outer coating of the guidewire is either hydrophobic (e.g., polytetrafluoroethylene (PTFE)) to reduce friction and facilitate advancement, or hydrophilic to enhance ‘slipperiness’ when in contact with blood [3,4,5,6]. Hydrophobic outer coatings provide varying degrees of electrical insulation depending on their composition. A number of coronary guidewires have been tested in vitro for use in trans coronary pacing (TCP) [2].

## 4. Trans Coronary Pacing

In the early days of percutaneous coronary revascularisation, procedural bradycardia was commonly observed due to myocardial ischemia resulting from prolonged angioplasty balloon inflation. This necessitated the placement of a right ventricular temporary pacing wire (TPW) [7]. However, this required central venous access which prolonged procedure time and increased the risk of complications (e.g., right ventricular perforation with tamponade, dysrhythmias, infection and vascular injury) [8].

The concept of cardiac pacing using a coronary artery guide wire was first investigated in 1984 by Chatelain et al. who demonstrated that short term cardiac pacing via a coronary guidewire was achievable and well tolerated in a swine model [9]. Later, Heinroth et al. performed a study to determine the effect of electrical insulation of the guidewire on pacing parameters during TCP [10]. Insulation using an angioplasty balloon catheter increased the success rate of myocardial capture from 77% to 100%. A series of other studies subsequently demonstrated the effectiveness and safety of TCP in animal models and in humans (Table 1) with very few adverse events (Table 2).

## 5. Clinical Applications of Trans Coronary Pacing

### 5.1. Pacing during Coronary Interventions

Although procedural bradycardia is relatively uncommon in contemporary PCIs, TCP remains a valuable but potentially overlooked option when transient cardiac pacing is indicated—for example in acute ST segment elevation myocardial infarction. Rotational atherectomy (RA) is utilised in complex PCI procedures to debulk coronary atheroma. The incidence of atrioventricular block and significant bradycardia is reported to be as high as 53% with this technique in the right coronary artery [24]. Transvenous pacing is generally used to treat bradyarrhythmia; however, recently, Iqbal et al. and Kusumoto et al. showed that pacing can be achieved safely using an insulated Rotawire [23,25].

Accurate coronary stent placement is crucial during a PCI, particularly in ostial and bifurcation lesions. Cardiac contraction can hinder precise stent positioning during implantation. However, rapid cardiac pacing can be used to reduce cardiac motion and Lasa et al. showed how rapid TCP was utilised in twenty-seven patients undergoing complex PCIs. In this series, 96% of patients had excellent angiographic results [17]. More recently, this technique has shown promise in ostial lesions [26] as well as in distal cap puncture in chronic total coronary occlusions [27].

### 5.2. Transcoronary Pacing to Assess Myocardial Viability Assessment

In patients undergoing PCI for symptomatic coronary artery disease, knowing whether myocardium is viable can be helpful in making decisions regarding revascularisation. This may be of particular importance in patients with left ventricular systolic dysfunction or prior myocardial infarction, as treating non-viable myocardium is unlikely to improve myocardial function or, more importantly, symptoms. Furthermore, these procedures can be complex and carry risks. Currently no technique provides live on-table data for myocardial viability; most patients must be referred for imaging (e.g., cardiac magnetic resonance (CMR)). Having access to on-table viability data may allow interventionalists to proceed with intervention immediately.

Myocardial viability assessment can be carried out with a range of imaging modalities (Table 3). One of the most commonly used techniques to assess viability is CMR imaging, which can evaluate myocardial scar burden, myocardial perfusion and contractile reserve [28]. However, CMR is relatively expensive, time consuming, delays revascularisation decisions (as it cannot be performed on table) and has some contraindications (e.g., patients with certain metallic implants, severe renal insufficiency or claustrophobia). Moreover, it may not be available in all centres. A cardiac PET scan is considered as the gold-standard imaging modality for myocardial viability assessment owing to high spatial and temporal resolutions combined with excellent sensitivity and negative predictive value. Myocardial perfusion (via N-13 ammonia) and metabolism [via fluorodeoxyglucose (FDG)] can also be evaluated. The switch of energy source to glucose from fatty acids by myocardial tissue in ischemic states forms the biochemical basis of using radio-labeled glucose i.e., FDG in PET scans. The indication of an enhanced uptake of FDG in regions of low perfusion indicates hibernating myocardium, while a concordant reduction in both perfusion and metabolism is suggestive of scarred myocardium [29]. Due to availability, cost and radiation exposure, PET scans are not routine [30].

Stress echocardiography, nuclear imaging and CMR imaging are commonly used to assess myocardial viability. Echocardiography, despite being readily available, can be limited by inter-observer variability and poor acoustic windows. Nuclear scans can overestimate scar burden due to low spatial resolution. None of these imaging-based investigations can be performed at the time of coronary angiography and intervention [29]. A real-time viability assessment at the time of angiography could be useful to enable rapid decision making during coronary intervention.

Ischaemia and infarction alter the biochemical and biophysical properties of the myocardium. Prolonged ischemia leads to intracellular acidosis, which in turn activates a cascade of events ultimately resulting in myocardial necrosis. Complete disruption of myocardial blood flow leads to infarction, which is not expected to regain contractile function if blood flow is restored (non-viable myocardium). However, chronic reduction in myocardial perfusion can trigger hibernation: a state in which the myocardial muscle is not contractile but can regain contractility following coronary revascularisation as myocardial ischaemia affects the electrophysiological properties of the heart [32,33]. Non-viable myocardium is electrically silent; therefore, an assessment of myocardial electrophysiology has been proposed as a potential method to assess viability.

Systematic experimental and clinical studies demonstrate that myocardial electrograms can differentiate non-viable from hibernating and normal myocardium on the basis of a lower amplitude in voltage, longer duration and fractionated electrograms [34,35,36,37,38,39,40,41,42,43,44]. Intracoronary electrograms (Ic ECG) (which will pick up myocardial signals) can be recorded with coronary guidewires. Once the guidewire is at the desired position within the artery local signals can be recorded [45,46,47].

Yajima et al. [48] and Abaci et al. [49] evaluated whether ST segment elevation on an Ic ECG, recorded following temporary balloon occlusion of a coronary artery during PCI after myocardial infarction, could act as a surrogate marker of myocardial viability. Intracoronary ECGs were recorded following a transient balloon occlusion of the coronary artery after advancing the tip of the coronary guidewire to a distal position. In patients undergoing an emergency PCI for acute myocardial infarction, Yajima et al. [48] demonstrated that increased ST segment elevation recorded after transient balloon occlusion of the reperfused infarct-related artery was associated with myocardial viability on subsequent thallium-201 myocardial scintigraphy. Abaci et al. [49] showed that ST segment elevation after transient balloon occlusion was predictive of myocardial viability in patients with recent non-Q wave myocardial infarction scheduled for a PCI. These studies suggested potential utility of the Ic ECG for viability assessment during index angiography [48,49].

Petrucci et al. [50] explored whether the amplitude of the Ic ECG, recorded using a standard coronary guide wire during a PCI in the absence of transient balloon occlusion during the measurement, is indicative of myocardial viability. The study recruited twenty-five patients with left ventricular systolic dysfunction who had an anterior STEMI more than 6 weeks previously and were scheduled for an elective PCI in a non-culprit coronary artery. Ic ECGs were recorded using a conventional coronary guidewire, electrically insulated with a micro catheter, leaving the distal 5 mm of the wire exposed. The Ic ECG recording site was assigned to corresponding segments on an MRI after analysing the wire position. The results indicated that the peak to peak amplitude of the Ic ECG signal could discriminate between viable and non-viable myocardium with a lower amplitude (mean 0.75 mV) recorded from non-viable myocardium as compared to a higher amplitude recorded from viable myocardium (mean 3.26 mV). In this study, a stepwise decline in amplitude was observed between Ic ECGs recorded from segments with viable, partially viable and non-viable myocardium (*p*-value < 0.0001).

The concept of utilising Ic electrophysiological parameters for myocardial viability assessment was further developed by O’Neill et al. who studied pacing parameters obtained during transient cardiac pacing via a conventional coronary guidewire [2]. The aim of this pilot study was to ascertain the potential utility of pacing threshold, impedance and R wave amplitude for the assessment of myocardial viability during PCI. Six patients scheduled for an elective PCI for significant stenosis of the left anterior descending artery were recruited. Forty-two myocardial segments were analysed using a cardiac MRI for viability. This study reported lower impedance of non-viable myocardium when compared to viable myocardium (222.3 Ohm vs. 304.8 Ohm, *p* value = 0.0091), with higher pacing thresholds observed in myocardial segments in which >50% late gadolinium enhancement (LGE) was present on CMR (3.9 V vs. 1.9 V, *p* value = 0.002). These findings suggested that the impedance and pacing threshold recorded during PCI via coronary guidewire can be utilised to differentiate viable from non-viable myocardium. Although the sample size of the patients was small, this study demonstrated the feasibility of utilising parameters measured during temporary coronary pacing for intra-procedural viable myocardial assessment [51].

The hypothesis of utilising electrophysiological parameters from trans coronary pacing to ascertain myocardial viability was tested in larger study of patients requiring a PCI to diseased left or right coronary arteries in both acute and elective settings [22]. A total of 65 patients participated in this study. Ic ECG amplitude and pacing threshold and impedance were recorded during trans coronary pacing in multiple coronary sites during a PCI. Myocardial viability was assessed for each corresponding myocardial segment using an ‘infarct score’ based on degree of late gadolinium enhancement on CMR. In contrast to previous studies, statistical analysis failed to demonstrate any significant relationship between R wave amplitude, threshold and impedance in healthy myocardium and infarcted myocardial tissue. These findings are contrary to previous studies which showed differences in the distribution and relation of parameters in healthy and scarred myocardium [2,45]. A relatively low number of segments with scarred myocardium in the latest study could be one potential reason for the discrepant results. However, the distribution of electrophysiological parameters in healthy myocardium showed a surprisingly wide variation. This raises the possibility that technical aspects of the procedure may have led to variability in electrical measurements and contributed to their failure to predict viability. It is likely that the exact position of the guidewire, the size of the branch into which the wire tip is advanced and the length of the distal tip exposed beyond the balloon catheter are of critical importance. Refining the technical aspects of Ic ECG recording, including a means of assessing contact or perhaps a specialised bipolar angioplasty, would need to be developed to refine the methodology and improve the sensitivity and specificity. If it is indeed possible to refine the technology, and larger studies show that trans coronary electrophysiological parameters show superiority over current methods of assessing viability, then it is possible that it could potentially replace the need for taking the patient off the table for non invasive viability assessments. This may well change the consent process as patients will need to be informed about the novel method of viability assessment and how it may impact on their procedure.

One surprising observation from this study is that it was often possible to capture myocardial segments that were demonstrated to have transmural infarction on the CMR. This implies that even these segments have viable myocardium. This phenomenon is not uncommonly observed in endocardial/epicardial mapping for ventricular tachycardia where these protected channels in scar tissue facilitate re-entry. Could this group of patients with scarred but excitable myocardium be at risk of ventricular arrhythmias? Could trans coronary electrophysiological parameters be used to predict risk? Further studies will be needed to investigate this.

## 6. Conclusions

Trans coronary pacing using a conventional coronary guidewire is feasible and safe (at least in small studies) and can be utilised in the management of transient bradycardia during PCI procedures. Electrical parameters recorded using a coronary guidewire, including transient ST segment elevation during balloon inflation or peak-to-peak voltage on Ic ECG, show promise in discriminating viable from non-viable myocardium in patients undergoing PCI. Although data from earlier studies had suggested that impedance and threshold values recorded during transient trans coronary pacing at the time of the PCI may be used in the assessment of myocardial viability, these findings have not been supported by the latest study [22]. The ability to discriminate between vessels supplying viable and non-viable myocardium in continuum of the procedure in the cardiac catheter laboratory would offer major advantages to patients scheduled for coronary angiography and ad hoc PCIs, particularly in health care systems with limited access to cardiac MRIs. Further studies are warranted in larger patient groups with improved technology and technique to determine the optimum approach to myocardial viability assessment using the coronary guidewire and determine the safety of the technique.

## Figures and Tables

**Table 1:** Trans coronary pacing studies in animal models and humans.

Study	Indication	No.	Paced Site (n)	Type and Position of Indifferent Electrode ***	Wire Used	Insulated with Catheter/Balloon	Max Output (In Volts)	Pacing Success	Comments
Chatelain, 1984 [9]	Bradycardia in PCI	Pigs 6		Subcutaneous needle	Metallic, Tinton Falls, NJ, USA	No	NS		-Paced at various levels without insulation. -Prolonged pacing led to thrombus formation.
Meier, 1985 [11]	Bradycardia in PCI	Human 22	25	Skin electrode, area 20 cm^2^	Guidewire, Schneider—Medintag, Zurich, Switzerland	No	NS	96%	LV pacing also performed with J-tip Cordis wires. One unsuccessful pacing site was completely infarcted.
De Le Serna, 1992 [12]	Bradycardia	Human 300	349	Skin electrode on L leg, area 20 cm^2^	Guidewire, Schneider—Medintag, Zurich, Switzerland	Yes	12	97%	In total, 2% needed therapeutic pacing.
Laird, 1993 [13]	Reliability during ischemia	Pigs 7	7	Skin needle, grounded at incision site	AC, Mountain view, CA, USA	Yes	10	100%	Intracoronary pacing capture was reliable during acute ischemia.
Mixon, 2004 (PCI) [14]	Bradycardia in PCI	Human 26	26	Steel monofilament suture 3–0 (B&S30)	Mod support Luge wire, Boston Scientific, Marlborough, MA, USA	Yes	12	100%	Aim was to demonstrate safety of TCP in treatment of bradycardia during PCI 53% (14) needed therapeutic pacing.
Heinroth, 2006 [15]	Bradycardia in PCI	Human 70	70	Skin electrode, area 100 cm^2^	Guidant, Guidant Corp, St. Paul, MN, USA	Yes	10	85.7%	In total, 4.3% needed therapeutic pacing.
Mixon, 2008 [16]	Bradycardia in PCI	Human 105	105	Steel monofilament suture needle	Luge Boston Scientific, USA	Yes	NS	96.2%	In total, 52% needed therapeutic pacing.
Lasa, 2009 [17]	Stent immobilisation	Human 27	27	Abbocath needle inserted into skin	NS	Yes	10	96%	Stent position better with rapid TCP.
Heinroth, 2009 [10]	Optimisation of TCP	Pigs	8	Skin electrode, area 100 cm^2^	Floppy tip, Boston Scientific, USA	Yes	10	100%	Correlation with TVP **.
Heinroth, 2011 [18]	Impact of guidewire insulation	Pigs 15		Skin electrode, area 100 cm^2^	Visionwire, Biotrinik, Germany Galeo floppy, Biotrinik, Berlin, Germany	No Yes	10		Insulation improved pacing efficacy from 77 to 100%.
Prondzinsky, 2012 [19]	Optimisation of TCP	Pigs	8	Intravascular electrodes	Floppy tip Boston Scientific, USA	Yes	10	100%	ICD * lead in aorta served as indifferent electrode. Electrical parameters comparable to skin electrode.
Heinroth, 2016 [20]	Use of guidewire as cathodal electrode	Pigs 16		Galeo floppy, Biotrinik, Germany Skin electrode. area 100 cm^2^	Visionwire, Biotrinik, Germany	No			Second uncoated guidewire (Galeo floppy) was utilised as indifferent electrode. Electrical parameters comparable to skin electrode in TCP setup.
O’Neill, 2017 [2]	Assess viability	Human 6	42	Skin electrode	BMW, Abbott Vascular, Plymouth, MN, USA	Yes	10	100%	Viable muscle differs in electrical parameters from scarred myocardium,
Heinroth, 2018 [21]	Reliability during ischemia and after PCI	Pigs 7		Skin electrode, area 100 cm^2^	Galeo Floppy, Biotrinik, Germany	Yes	10	100%	TCP can be performed reliably during ischaemia,
Javid, 2023 [22]	Assess myocardial viability	Human 65	369	Skin electrode	Sion Blue, Asahi Intecc, Sagamihara, Japan	Yes	10		Small number of infarcted segments, No correlation between EP parameters and viability on MRI. Can also pace in full-thickness scars,
Iqbal B., 2023 [23]	Bradycardia in rotational atherectomy	Human 137		Skin needle	Rotawire, Boston Scientific, USA	Yes	NS	91.5%	Inability to pace in areas with large infarcts which may have been contributed by lipid-based flush solution with electrical insulation.

NS: not stated. * ICD: Implantable cardioverter defibrillator; ** TVP: Transvenous pacing; *** Various electrodes were used in studies. Some used needles through the skin and others used self-adhesive skin patch electrodes to complete unipolar settings circuit for pacing. The details of the electrodes were not described in depth in all studies.

**Table 2:** Potential complications of trans coronary pacing [8,14,15].

Adverse Features of Transcoronary Pacing	Treatment
Coronary spasm	Administration of coronary vasodilators, e.g., intracoronary nitrates Keep pacing for short periods only Reduce pacing output voltage
Skin irritation	Use skin electrodes with larger surface area Anaesthetise the skin with local anaesthetic spray to reduce irritation and discomfort
Diaphragmatic Stimulation	Change position of the wire
Atrial pacing	Insulate by advancing balloon or micro catheter to avoid electrical capture of atria
Thrombosis at guidewire tip	Heparin given initially and further according to ACT *
Dissection/perforation of coronary artery	Careful wire manipulation under fluoroscopy; avoiding deep advancement of balloon or micro catheter to small branches

* ACT: activated clotting time.

**Table 3:** Imaging Modalities to assess myocardial improvement after revascularisation.

Method	Patients, n	Sensitivity, %	Specificity, %	PPV, %	NPV, %
Db-echo	1421	80	78	85	83
^201^Tl	858	87	54	67	79
^99m^Tc	488	83	65	74	76
PET-^18^F-FDG	598	92	63	74	87
LGE-CMR	331	95	51	69	90
Db-CMR	247	81	91	93	75

Db-CMR indicates dobutamine cardiovascular magnetic resonance; Db-Echo, dobutamine echocardiography; LGE-CMR, late gadolinium-enhanced cardiovascular magnetic resonance; PET-18F-FDG, positron emission tomography–fluorodeoxyglucose; NPV, negative-predictive value; PPV, positive predictive value; ^99m^Tc, technetium-99m; and ^201^Tl, thallium-201 [31].

## Data Availability

Not applicable.

## Abbreviations

TCP	Trans coronary pacing
Ic	Intracoronary
CMR	Cardiovascular magnetic resonance imaging
DSE	Dobutamine stress echocardiography
CT	computed tomography
PCI	Percutaneous coronary intervention
LGE	late gadolinium enhancement
MI	Myocardial infarction
VT	Ventricular tachycardia
CTO	Chronic total occlusion
